# Supplementary material for: Reduced Toxicity of Centruroides vittatus (Say, 1821) May Result from Lowered Sodium β Toxin Gene Expression and Toxin Protein Production
Source: Toxins (Basel). 2021 Nov 22;13(11):828. doi: 10.3390/toxins13110828 (PMC8619477; doi:10.3390/toxins13110828)
Supplement: Supplementary file 1 [file toxins-13-00828-s001.zip › toxins-1337038-supplementary.pdf]

Supplementary Materials: Reduced Toxicity of *Centruroides vittatus* (Say, 1821) May Result from Lowered Sodium β Toxin Gene Expression and Toxin Protein Production

Aimee Bowman, Chloe Fitzgerald, Jeff F. Pummill, Douglas D. Rhoads and Tsunemi Yamashita

Table S1. Putative *C. vittatus* Sodium Beta Toxin polypeptide sequence diversity from a survey of 10 population groups identified in a *C. vittatus* phylogeographic analysis. Also included are CsEv3b and CvIV4 toxin sequences and additional *C. sculupturatus* Beta toxin sequences obtained through a Cloning experiment. Cysteine residues are highlighted in green. .

```
                    1        10        20        30        40        50        60        70        80        90       100       110       120
                    |        |         |         |         |         |         |         |         |         |         |         |         |
           CsEv3b   KEGYLVNKSTGCKYGCLKLGENEGCDKECKAKNQGGSYG-YCY-AFACWCEGLPESTPTYPLPNKSCGKK----------------------------------------------------
            CvIV4   KKDGYPVEHS-GCKYTCWK---NEYCDKV-CK--DLKGEGG-YCYINLTCWCTGLPDNVPLKT--NQRCNGKRK------------------------------------------------
      CsCNaTBet01   REGYLVRKSDNCKHGCIPGIDEDYCGDICKKRNRGGKKGWCQKYGCWCTGMPESTQTYPIPGKPCSD-------------------------------------------------------
      CsCNaTBet03   REGYLVRKSDNCKHGCIPGIDEDYCGDICKKRNRGGKKGWCQKYGCWCTGMPESTQTYPIPGKPCSD-------------------------------------------------------
CsCNaTBet04   KEGYLAIRGCIYSCVTDRECETECKKYGGKIGFCRFLCYCEGLPKSVPTLPKPGKKCAIPGFSSITSEFAAACRSTIWESSQRVGCIA----------------------------------
 CviNaTBet_BCR_01   KEGFLALRGCYAVV*RIGSAKQNARSTEEKLDFADFLRAIAKVCPKVYRLCPNLVENAPYRASP----------------------------------------------------------
 CviNaTBet_BCR_02   KEGFLAMRGCYTVA*RIGSAKQNARSTEEKLDFADFLRAIAKVCPKVYRLCPNLVENAPYRASP----------------------------------------------------------
 CviNaTBet_BCR_03   PEGWDAYLECSIVPPK*RIGSAKQNARSTEEKLDFADFLRAIAKVCPKVYRLCPNLVENAPYRASP--------------------------------------------------------
 CviNaTBet_BCR_04   KEGYLAIRGCIYSCVTDRECETECKKYGGKIGFCRFLTCYCEGLPKSVPTVPKPGRKCAIPGFS----------------------------------------------------------
 CviNaTBet_BCR_05   KEGYLAIRGCIYSCVTDRECETECKKYGGKIGFCRFLTCYCEGPPKSVPTVPKPGRKCAIPGFSSITSEFAAACRSTIWESSQRVGCIA---------------------------------
 CviNaTBet_BCR_06   KDGYLALRGCNAVA*RIGSAEQNARSTEEKLDFADFLRAIAKVCPKVYRLCPNLVENAPYRASP----------------------------------------------------------
 CviNaTBet_BCR_07   KDGILP*GFYNAAA*RIGSAKQNARSTEEKLDFADFLLAIAKVCPKVYRLCPNLVENAPYRASP----------------------------------------------------------
 CviNaTBet_BCR_08   KEGYLVNKKTGCKYNCMLKIGDSHCDKECKAPNQGGSYGYCFKLGCWCEGLPESTPTYPLPDKSCSG-------------------------------------------------------
 CviNaTBet_BCR_09   REGYLVNKKT----------------------------------------------------------------------------------------------------------------
 CviNaTBet_BCR_10   KEGYLVKKSDGCKYGCVMLFGDSNCDMECKAPNQGGQKGWCYAFACWCTGMPESAQVYPSDKSCGKK-------------------------------------------------------
 CviNaTBet_BCR_11   KEGYLVKKSDNCKHGCIPGFDEDYCDDICKTRNRGGKKGWCQKYGCWCTGMPESTQTYPIPGKPCSS-------------------------------------------------------
 CviNaTBet_BCR_12   REGYQISGSDLAPNPVTQQLMNLGATIYFNHRPENVRDASVVVVSSAISADNPPL-------------------------------------------------------------------
 CviNaTBet_BCR_13   KEGYLVNKKT----------------------------------------------------------------------------------------------------------------
 CviNaTBet_BCR_14   -------------------------KEGYLAKNQGGEKG-WCK-NFGCWCTGMPESTPTWPI-GKPCSGK----------------------------------------------------
 CviNaTBet_BCR_15   KEGYLAKKGDGCKYGCTPYFGDEGCDKECKAKNQGGEKG-WCK-NFGCWCTGMPESTPTWPI-GKPCSGK----------------------------------------------------
  CviNaTBet_HT_01   KEGYLVNKSDGCKYGCVMLIGDEGCNKECKAPNQGGTKGWCYAFGCWCTGMPESTQVYPLPNKSCGKK------------------------------------------------------
  CviNaTBet_HT_02   REGYLAIRGCIYSCVTDRECETECKKYGGKIGFCRFLTCYCEGLPKSVPTVPKPGRKCA---------------------------------------------------------------
  CviNaTBet_HT_03   REGYLVNKSDGCKYGCVMLIGDEGCNKECKAPNQGGTKGWCYAFGCWCTGMPESTQVYPLPNKSCGKK------------------------------------------------------
  CviNaTBet_HT_04   REGYLVRKSSNCKDGCIPGIDEDYCDDRCKARNQGGKKGWCQKYGCWCTGMPESTQTYPIPGKPCSD-------------------------------------------------------
  CviNaTBet_HT_05   REGYLAIRGCIYSCVTDRECETECKKYGGKIGFCRFLTCYCEGLPKSVPTLPKPGRKCA---------------------------------------------------------------
  CviNaTBet_HT_06   KEGYLAIRGCIYSCVTDRECETECKKYGGKIGFCRFLTCYCEGLPKSVPTLPKPGRKCA---------------------------------------------------------------
  CviNaTBet_HT_07   REGYLYAVGGNDGSSSLNTVERYNPRHNKWMLVTSMTLRRSSVGVAVVDSPCMELILTRKSGQ-----------------------------------------------------------
  CviNaTBet_HT_08   REGYLVNKKTGCKYGCTPKLGDDHCDKECKAPNQGGKKGWCKNFGCWCTGMPESTQTWPIPGKSCSS-------------------------------------------------------
  CviNaTBet_HT_09   REGYLVKKSDGCKYGCTPKLGDDHCDKECKAPNQGGKKGWCKNFGCWCTGMPESTQTWPIPGKSCSR-------------------------------------------------------
CviNaTBet_Chin_01   KEGYLVKKSDGCKYGCVMLIGDSNCDMECKAPNQGGQKGWCYAFGCWCTGMPESTQVYPLPGKSCGKK------------------------------------------------------
CviNaTBet_Chin_02   KEGYLVKKSDGCKYGCVMLIGDSNCDMECKAPNQGGQKGWCYAFACWCTGMPESAQVYPS-DKSCGKK------------------------------------------------------
CviNaTBet_Chin_03   REGYLA--IRGCIYSCV---TDRECETECK--KYGGKIGFCRFLTCYCEGLPKSVPTLPKPGRKCA--------------------------------------------------------
CviNaTBet_Chin_04   REGYLVKKSDGCKYGCVMLIGDSNCDMECKA-------------------------------------------------------------------------------------------
CviNaTBet_Chin_05   KEGYLVRKSDNCKHGCIPGIDEDFCDDICKARNRGGKKGWCQKYGCWCTGMPESTQTYPIPGKPCSS-------------------------------------------------------
CviNaTBet_Chin654   KEGYLAIKTTCCKYTRMT---DRVCIAECK--KNGGKTG-FCQ-FLGCYCLDLPESAPTWPIHGKPCSGK----------------------------------------------------
CviNaTBet_AgSp_01   REGYLAIRGCIYSCVADRECETECKKYGGKTGFCRFLTCYCEGLPKSVPTLPKPGRKCA---------------------------------------------------------------
```

```
CviNaTBet_AgSp_02   KEGYLAIRGCIYSCVTDRECETECKKYGGKIGFCRFLTCYCEGLPKSVPTVPKPGRKCA--------------------------------------------------------------
CviNaTBet_AgSp_03   REGYLVKKSDNCKHGCIPGFDEDYCDDICKTRNRGGKKGWCQKYGCWCTGMPESTQTYPIPGKPCSS-------------------------------------------------------
CviNaTBet_AgSp_04   KEGYLVKKSDNCKHGCIPGFDEDYCDDICKTRNRGGKKGWCQKYGCWCTGMPESTQTYPIPGKPCSS-------------------------------------------------------
CviNaTBet_AgSp_05   REGYLVNKSDGCKYGCVMLIGDEGCNKECKAPNQGGTKGWCYAFGCWCTGMPESTQVYPLPNKSCGKK------------------------------------------------------
CviNaTBet_AgSp667   KEGYLAIR--GCIYSCVT---DRECETECK--KYGGKIG-FCR-FLTCYCEGLPKSVPTVPKPGRKCA------------------------------------------------------
CviNaTBet_AgSp668   KEGYLVKKSDGCKYGCTPYFGDEGCDKECKAKNQGGEKG-WCK-NFGCWCTGMPESTPTWPI-DKTCSKK----------------------------------------------------
CviNaTBet_AgSp_06   REGYLVKKSDNCKHGCIPGFDEDYCD-DICKTRNRGGKKG-WCQ-KYGCWCTGMPESTQTYPIPGKPCSS----------------------------------------------------
CviNaTBet_AgSp_07   REGYLAIR--GCIYSCVA---DRECETECK--KYGGKTG-FCR-FLTCYCEGLPKSVPTLPKPGRKCA------------------------------------------------------
CviNaTBet_AgSp_08   KEGYLVKKSDNCKHGCIPGFDEDYCD-DICKTRNRGGKKG-WCQ-KYGCWCTGMPESTQTYPIPGKPCSS----------------------------------------------------
CviNaTBet_AgSp_09   REGYLVNKSDGCKYGCVMLIGDEGCNKECKAPNQGGTKG-WCY-AFGCWCTGMPESTQVYPLPNKSCGKK----------------------------------------------------
CviNaTBet_AgSp_10   KEGFWRSGAVIQLRDG*GVLNRLQEVRRKNWILPIFTCYC*GLPKSVPTVPKPGRKCAIPGFSSITIEFAAACMSTLWESSQRVGCIA----------------------------------
CviNaTBet_AgSp_11   KEGYLAMRGCIYSCVTDRECETECKKYGGKIGFCRFLTCYCEGLPKSVPTVPKPGRKCAIPGFSSITSEFAAACRSTIWESSQRVGCIA---------------------------------
 CviNaTBet_LBR_01   REGYLVKKSDGCKYGCVMLIGDSNCDMECKAPSQGGQKGWCYAFGCWCTGMPEGTQVYPLPGKSCGKK------------------------------------------------------
 CviNaTBet_LBR_02   REGYLVKKSDGCKYGCVMLIGDSHCDTECKAPNQGGKKGWCYALGCWCTGMPESTQVYPLPDKSCGKK------------------------------------------------------
 CviNaTBet_LBR_03   REGYLVNKSDGRKYGCVMLIGDEGCNKECKAPNQGGTKGWCYAFGCWCTGMPESTQVYPLPNKSCGKK------------------------------------------------------
 CviNaTBet_LBR_04   KEGYLVRKSDNCKDGCIPGIDEDYCDDRCKARNQGGKKGWCQKYGCWCTGMPESTQTYPIPGKPCSS-------------------------------------------------------
 CviNaTBet_LPb_01   KEGYQVNKSDGCKYGCVMLIGDEGCNKECKAPNQGGTKGWCYAFGCWCTGMPESTQVYPLPNKSCGKK------------------------------------------------------
 CviNaTBet_LPb_02   KEGYLAIRGCIYSCLTDRECETECKKYGGKIGFCRFLTCDCEGLPKSVPTVPKPGRKCA--------------------------------------------------------------
CviNaTBet_LPb3066   REGYLVKKSDGCKYGCVMLIGDSHCDTECKAPNQGGKKGWCYALGCWCTGMPESTQVYPLPDKSCGKK------------------------------------------------------
 CviNaTBet_LPb_03   KEGYLVNKKTGCKYNCMLKIGDSHCDKECKAPNQGGSYGYCFKLGCWCEGLPESTPTYPLPDKSCSG-------------------------------------------------------
 CviNaTBet_LPb_04   REGYLVNKKTGCKYNCMLKIGDSHCDKECKAPNQGGSYGYCFKLGCWCEGLPESTPTYPLPDKSCSG-------------------------------------------------------
 CviNaTBet_BMe_01   KEGYLALR--GCIYSCVT---DREYEAECK--KIGGKIGIWC--FFTSSCEGMSKGGQPVPQLGR---------------------------------------------------------
CviNaTBet_PaDu_01   KEGCLALRGLYTVG*RIGSAEQNARVRIKNWILPISYLLLLGSAQNSPTLPKLGRKCAIPGFSSITIEFAATCRPPLWESSQRVGCIPCLFYSV**R-------------------------
CviNaTBet_PaDu_01   KEGYLAIRGCIYSCVTDRERETECKKYGGKIGFCRFLTCYCEGLPKSVPTLPKPGRKCAIPGFSSITSEFAAACRSTIWESSQRVGCIA---------------------------------
CviNaTBet_PaDu_02   KEGFLAMRGCSTVA*RIGSAKQNARSTEEKLDFADFLRAIAKVCPKVYRLCPNLVENAPYRASPQSLVNSRPPAGRPYGRAPNALDA-----------------------------------
CviNaTBet_PaDu_03   KEGYLVNKSDGCKYGCVMLIGDEGCNKECKAPNQGGTKGWCYAFGCWCTGMPESTQVYPLPNKSCGKK------------------------------------------------------
CviNaTBet_PaDu_04   KEGYLAIRGCIYSCVTDRECETECKKYGGKIGFCRFLTCYCEGLPKSVPTVPKPGGKCA--------------------------------------------------------------
CviNaTBet_PaDu_05   KEGYLVNKSNGCKYGCTPKLGDDHCDKECKARNQGGKKGWCKNFGC----------------------------------------------------------------------------
CviNaTBet_Kisa_01   KEGYLVNKSDGXIRLRDVDWRRGLQ-------------------------------------------------------------------------------------------------
CviNaTBet_Kisa_02   KEGYLVRKSDNCKHGCIPGIDEDFCDDICKARNRGGKKGWCQKYGCWCTGMPESTQTYPIPGKSCSS-------------------------------------------------------
CviNaTBet_Kisa_03   KEGYLVKKSDGCKYGCTPYFGDEGCDKECKAKNQGGEKGWCKNFGCWCTGMPESTPTWPI-DKTCSKK------------------------------------------------------
CviNaTBet_Kisa_04   REGYLVKKSDGCKYGCVMLIGDSNCDMECKAPSQGGQKGWCYAFGCWCTGMPEGTQVYPLPGKSCGKK------------------------------------------------------
CviNaTBet_Kisa_05   KEGYAIRGCIYSCVTDRECETECKKYGGKIGFCRFLTCYCEGLPKSVPTVPKPGRKCA---------------------------------------------------------------
  CviNaTBet_KS_01   KEGFWRSGAVIQLRDG*GVLNRLQEVRRKNWILPIFTCYC*GLPKSVPTVPKPGRKCAIPGFSSITIEFAAACMSTLWESSQRVGCIA*VFYSVT*IA------------------------
  CviNaTBet_KS_02   KEGYLAMRGCIYSCVTDRECETECKKYGGKIGFCRFLTCYCEGLPKSVPTVPKPGRKCAIPGFSSITSEFAAACRSTIWESSQRVGCIA---------------------------------
  CviNaTBet_KS_03   KEGYLAIRGCIYSCVTDRECETECKKYGGKIGFCRFLTCYCEGLPKSVPTVPKPGRKCAIPGFSSITSEFAAACRSTIWESSQRVGCIA---------------------------------
  CviNaTBet_KS_04   KEGYLAIRGCIYSCVTDRECETECKKYGGKIGFCRFLTCYCEGLLKSVPTVPKPGRNAPYRASPQSLVNSRPPAGRPYGRAPNALDA-----------------------------------
  CviNaTBet_KS_05   REGYLVNKKTGCKYNCMLKIGDSHCDKECKAPNQGGSYGYCFKLGCWCEGLPESTPTYPLPDKSCSG-------------------------------------------------------
 CviNaTBet_Ha1210   KDGYLVKKSDGCKYGCVMLIGDSNCDMECKAPNQGGQKGWCYAFGCWCTGMPESTQVYPLPGKSCGKK------------------------------------------------------
 CviNaTBet_SCv_01   KEGYLVNKSDGCKYGCVMLIGDEGCNKECKAPNQGGTKGWCYAFGCWCTGMPESTQVYPLPNKSCGKK------------------------------------------------------
 CviNaTBet_SCv_02   KEGYLVNKSNGCKYGCTPKLGDDHCDTECKARNQGGKKGWCKNFGCWCTGMPESTQTYPIPGKPCSS-------------------------------------------------------
```

Table S2. Normalized relative expression results from eight sodium beta toxins and four reference genes. a) One way ANOVAs from RT-qPCR expression values for males and females with eight sodium beta toxin genes and standardized with four reference genes.

A) One way ANOVA.

Males

**SUMMARY**

Groups	Count	Sum	Average	Variance
AgSp_667	8	19.17065	2.396332	11.8237
AgSp_668	8	9.526841	1.190855	0.487886
CsEv3b	8	37.64851	4.706064	9.340745
CvIV4	8	27.24754	3.405943	8.519668
Ha_1210	8	38.62098	4.827623	18.08439
LPB_03	8	32.07183	4.008978	26.13679
Chin_654	8	10.68958	1.336198	1.295146
HT_09	8	15.02775	1.878469	5.43295

**ANOVA**						
Source of Variation	*SS*	*df*	*MS*	*F*	*P-value*	*F crit*
Between Groups	120.7152	7	17.24502	1.700666	0.127587	2.178156
Within Groups	567.8489	56	10.14016			
Total	688.5641	63				

Females

**SUMMARY**				
Groups	*Count*	*Sum*	*Average*	*Variance*
AgSp_667	13	30.37719	2.336707	2.407683
AgSp_668	13	19.60685	1.508219	0.987705
CsEv3b	13	33.25781	2.558293	2.03941
CvIV4	13	25.49047	1.960806	1.037641
Ha_1210	13	59.96065	4.612357	9.164563
LPB_03	13	50.22582	3.863524	12.43038
Chin_654	13	21.50034	1.653872	0.500684
HT_09	13	25.16963	1.936125	1.046539

**ANOVA**						
Source of Variation	*SS*	*df*	*MS*	*F*	*P-value*	*F crit*
Between Groups	112.274	7	16.03914	4.332764	0.00033	2.106465
Within Groups	355.3752	96	3.701825			

Total	467.6492	103

b) Tukey's pairwise test for males and females.

**Males**				
**Na Tox Gene**	**diff**	**lwr**	**upr**	**p adj**
Chin_654-LPB_03	-2.6728	-7.6854	2.3399	0.7005
AgSp_667- LPB_03	-1.6126	-6.6253	3.4000	0.9706
AgSp_668- LPB_03	-2.8181	-7.8308	2.1945	0.6423
HT_09- LPB_03	-2.1305	-7.1431	2.8821	0.8801
CsBeta- LPB_03	0.6971	-4.3155	5.7097	0.9998
CvIV4- LPB_03	-0.6030	-5.6157	4.4096	0.9999
Ha1210- LPB_03	0.8186	-4.1940	5.8313	0.9995
AgSp_667- Chin_654	1.0601	-3.9525	6.0728	0.9976
AgSp_668- Chin_654	-0.1453	-5.1580	4.8673	1.0000
HT_09- Chin_654	0.5423	-4.4704	5.5549	1.0000
CsBeta- Chin_654	3.3699	-1.6428	8.3825	0.4174
CvIV4- Chin_654	2.0697	-2.9429	7.0824	0.8951
Ha1210- Chin_654	3.4914	-1.5212	8.5041	0.3716
AgSp_668- AgSp_667	-1.2055	-6.2181	3.8072	0.9946
HT_09- AgSp_667	-0.5179	-5.5305	4.4948	1.0000
CsBeta- AgSp_667	2.3097	-2.7029	7.3224	0.8294
CvIV4- AgSp_667	1.0096	-4.0030	6.0222	0.9982
Ha1210- AgSp_667	2.4313	-2.5813	7.4439	0.7897
HT_09- AgSp_668	0.6876	-4.3250	5.7002	0.9999
CsBeta- AgSp_668	3.5152	-1.4974	8.5278	0.3630
CvIV4- AgSp_668	2.2151	-2.7975	7.2277	0.8574
Ha1210- AgSp_668	3.6368	-1.3759	8.6494	0.3204
CsBeta- HT_09	2.8276	-2.1850	7.8402	0.6384
CvIV4- HT_09	1.5275	-3.4852	6.5401	0.9782
Ha1210- HT_09	2.9492	-2.0635	7.9618	0.5883
CvIV4-CsBeta	-1.3001	-6.3128	3.7125	0.9915
Ha1210-CsBeta	0.1216	-4.8911	5.1342	1.0000
Ha1210-CvIV4	1.4217	-3.5910	6.4343	0.9855

**Females**				
**Na Tox Gene**	**diff**	**lwr**	**upr**	**p adj**
Chin_654-LPB_03	-2.2097	-4.5482	0.1289	0.0782
AgSp_667-LPB_03	-1.5268	-3.8654	0.8117	0.4721
AgSp_668-LPB_03	-2.3553	-4.6939	-0.0167	0.0471
HT_09- LPB_03	-1.9274	-4.2660	0.4112	0.1861
CsBeta-LPB_03	-1.3052	-3.6438	1.0333	0.6681
CvIV4-LPB_03	-1.9027	-4.2413	0.4358	0.1992
Ha1210-LPB_03	0.7488	-1.5897	3.0874	0.9745
AgSp_667-Chin_654	0.6828	-1.6557	3.0214	0.9849
AgSp_668-Chin_654	-0.1457	-2.4842	2.1929	1.0000
HT_09-Chin_654	0.2823	-2.0563	2.6208	0.9999
CsBeta-Chin_654	0.9044	-1.4341	3.2430	0.9305
CvIV4-Chin_654	0.3069	-2.0316	2.6455	0.9999
Ha1210-Chin_654	2.9585	0.6199	5.2970	0.0040
AgSp_668-AgSp_667	-0.8285	-3.1670	1.5101	0.9558
HT_09-AgSp_667	-0.4006	-2.7391	1.9380	0.9995
CsBeta-AgSp_667	0.2216	-2.1170	2.5601	1.0000
CvIV4-AgSp_667	-0.3759	-2.7145	1.9627	0.9996
Ha1210-AgSp_667	2.2757	-0.0629	4.6142	0.0624
HT_09-AgSp_668	0.4279	-1.9107	2.7665	0.9992
CsBeta-AgSp_668	1.0501	-1.2885	3.3886	0.8590
CvIV4-AgSp_668	0.4526	-1.8860	2.7911	0.9988
Ha1210-AgSp_668	3.1041	0.7656	5.4427	0.0020
CsBeta-HT_09	0.6222	-1.7164	2.9607	0.9913
CvIV4-HT_09	0.0247	-2.3139	2.3632	1.0000
Ha1210-HT_09	2.6762	0.3377	5.0148	0.0135
CvIV4-CsBeta	-0.5975	-2.9360	1.7411	0.9932
Ha1210-CsBeta	2.0541	-0.2845	4.3926	0.1287
Ha1210-CvIV4	2.6516	0.3130	4.9901	0.0150

Table S3. Blast alignments with NCBI accession numbers from 2133 toxin gene sequences queried against a female *C. vittatus* transcriptome assembly.

**NaScTx (19):**
JF938594.1 Centruroides vittatus alpha-toxin (IV4) mRNA, partial cds protein=Na+-channel blocking toxin
L05062.1_cds_AAA28287.1_1 Na+-channel blocking toxin
XM_023360957.1_cds_XP_023216725.1_1 putative sodium channel alpha-toxin Acra5 isoform X2
NW_019384318.1_cds_XP_023222714.1_59 sodium/glucose cotransporter 1-like
NW_019384389.1_cds_XP_023215424.1_20 amiloride-sensitive sodium channel subunit gamma-like
NW_019385320.1_cds_XP_023238892.1_14 sodium-dependent phosphate transport protein 1, chloroplastic-like
NW_019384764.1_cds_XP_023228501.1_35 alpha-toxin CvIV4-like
gi|324035393|gb|HQ262494.1| Centruroides suffusus suffuses beta-neurotoxin CssIX precursor, mRNA, complete cds
NW_019385573.1_cds_XP_023242001.1_7 alpha-toxin Cn12-like
NW_019385668.1_cds_XP_023242920.1_4 alpha-toxin CsE5
XM_023353973.1_cds_XP_023209741.1_1 ikitoxin-like
XM_023374868.1_cds_XP_023230636.1_1 alpha-toxin Cn12
XM_023367539.1_cds_XP_023223307.1_1 beta-toxin Im-2-like
NW_019385453.1_cds_XP_023240702.1_3 putative beta-neurotoxin RjAa1
NW_019385910.1_cds_XP_023209523.1_6 toxin Acra I-2-like]
NW_019384864.1_cds_XP_023230636.1_4 alpha-toxin Cn12
EU159284.1_cds_ABX76757.1_1 neurotoxin LmNaTx9 precursor
XM_023355851.1_cds_XP_023211619.1_1 neurotoxin Cex9-like
NW_019384332.1_cds_XP_023230234.1_30 lipolysis-activating peptide 1-alpha chain-like
**Metalloproteases (10),**
NW_019384931.1_cds_XP_023232100.1_17 astacin-like metalloprotease toxin 4
NW_019384931.1_cds_XP_023232109.1_24 astacin-like metalloprotease toxin 5
NW_019384931.1_cds_XP_023232116.1_31 astacin-like metalloprotease toxin 5 isoform X5

NW_019384470.1_cds_XP_023218978.1_2 astacin-like metalloprotease toxin 2
NW_019384854.1_cds_XP_023230424.1_1 astacin-like metalloprotease toxin 1
NW_019384400.1_cds_XP_023215965.1_33 astacin-like metalloprotease toxin 5
NW_019384816.1_cds_XP_023229533.1_12 astacin-like metalloprotease toxin 3
NW_019385291.1_cds_XP_023238474.1_3 astacin-like metalloprotease toxin 5
XM_023376335.1_cds_XP_023232103.1_1 astacin-like metalloprotease toxin 4
XM_023374656.1_cds_XP_023230424.1_1 astacin-like metalloprotease toxin 1

KScTx (6)

XM_023364460.1_cds_XP_023220228.1_1 potassium channel toxin TdiKIK isoform X2
XM_023383132.1_cds_XP_023238900.1_1 toxin KTx8-like
NW_019385535.1_cds_XP_023241648.1_10 potassium channel toxin gamma-KTx 1.1-like
AF288205.1_cds_AAG38523.1_1 ergtoxin precursor
gi|2959749|emb|AJ224689.1| Centruroides noxius mRNA for cobatoxin 1, complete CDS
AF288205.1_cds_AAG38523.1_1 Centruroides noxius ergtoxin-like protein 1 (Erg1) mRNA, complete cds

Phospholipases (2),

NW_019384400.1_cds_XP_023215959.1_27 cytosolic phospholipase A2-like isoform X1
NW_019384816.1_cds_XP_023229534.1_2 group XIIA secretory phospholipase A2-like

IGFBP (2),

NW_019384586.1_cds_XP_023223251.1_8 venom protein 302-like
NW_019384389.1_cds_XP_023215406.1_16 venom protein 30.1-like

Host Defense Peptides (2),

KU569300.1_cds_AMX81487.1_1 venom toxin meuVNP3
KU513835.1_cds_AMX81465.1_1 venom toxin meuEnz25

Serpins (1).

NW_019384413.1_cds_XP_023216515.1_33 protein=serpin B3-like

Table S4. Toxin proteins identified from 273 total proteins from a proteomics analyses for four females and three males. Quantitative Value (NSAF) are shown.

**Specimen**			**F1**		**F2**		**F3**		**F4**		**M1**		**M2**		**M3**	
**Identified Proteins (273)**	**Accession Number**	**Molecular Weight**	**Spectral Counts**	**NSAF**	**Spectral Counts**	**NSAF**	**Spectral Counts**	**NSAF**	**Spectral Counts**	**NSAF**	**Spectral Counts**	**NSAF**	**Spectral Counts**	**NSAF**	**Spectral Counts**	**NSAF**
alpha-toxin CvIV4-like [*Centruroides sculpturatus*]	XP_023228501.1	10 kDa	2	0.29%	3	0.56%	3	0.70%	1	0.25%	5	0.94%	3	0.51%	2	0.56%
alpha-toxin, partial [*Centruroides vittatus*]	AEI61921.1 (+1)	11 kDa	3	0.0075	4	0.0065	6	0.0121	3	0.0064	3	0.0081	2	0.0044	3	0.0073
alpha-toxin CvIV4 [*Centruroides sculpturatus*]	XP_023238166.1	9 kDa	1	0.29%	5	0.95%	2	0.47%	1	0.25%	1	0.32%	2	0.52%	1	0.28%
potassium channel toxin TdiKIK isoform X2 [*Centruroides sculpturatus*]	XP_023220228.1	16 kDa	4	0.34%	3	0.11%	2	0.27%	3	0.29%	6	0.55%	5	0.30%	2	0.33%
RecName: Full=Toxin Cg2	P60163.1	8 kDa	na	na	na	na	3	0.87%	1	0.30%	na	na	na	na	1	0.35%
ikitoxin-like [*Centruroides sculpturatus*]	XP_023218231.1 (+1)	11 kDa	na	na	na	na	2	0.38%	1	0.20%	na	na	na	na	1	0.23%
scorpine-like peptide Tco 41.46-2 [*Centruroides sculpturatus*]	XP_023220230.1	10 kDa	1	0.28%	2	0.36%	2	0.45%	2	0.48%	2	0.60%	2	0.49%	1	0.27%
arachidonate 5-lipoxygenase-like, partial [*Centruroides sculpturatus*]	XP_023217790.1	26 kDa	na	0.00%	1	0.07%	1	0.09%	2	0.19%	1	0.12%	1	0.10%	1	0.11%
RecName: Full=Alpha-toxin Cn12	P63019.1 (+2)	7 kDa	na	na	na	na	1	0.29%	1	0.31%	na	na	na	na	2	0.70%
beta-toxin Im-2-like [*Centruroides sculpturatus*]	XP_023223307.1	9 kDa	na	na	na	na	1	0.23%	na	na	na	na	na	na	2	0.55%
ras-related C3 botulinum toxin substrate 1 [*Centruroides sculpturatus*]	XP_023223053.1	22 kDa	3	0.38%	3	0.25%	2	0.20%	na	na	5	0.68%	3	0.33%	1	0.12%
ras-related C3 botulinum toxin substrate 1-like [*Centruroides sculpturatus]*	XP_023232971.1	23 kDa	2	0.24%	0	0.00%	1	0.10%	1	0.10%	3	0.39%	2	0.21%	1	0.12%
beta-toxin CeII8-like [*Centruroides sculpturatus*]	XP_023237823.1	10 kDa	na	na	na	na	1	0.23%	1	0.25%	na	na	na	na	na	na
RecName: Full=Alpha-toxin CsE5	P46066.1 (+6)	7 kDa	na	na	na	na	1	0.31%	1	0.33%	na	na	na	na	1	0.37%
beta-neurotoxin CssIX precursor [*Centruroides suffusus suffusus*]	ADY17426.1 (+2)	9 kDa	na	na	na	na	1	0.24%	na	na	na	na	na	na	na	na
RecName: Full=Toxin Cn11	P58296.1	7 kDa	1	0.39%	1	0.25%	1	0.31%	2	0.66%	1	0.42%	1	0.34%	2	0.75%
RecName: Full=Toxin II.9	P60264.1	3 kDa	3	2.52%	7	3.81%	na	na	na	na	3	2.72%	na	na	na	na
CviNaTBet_Ha1210	MT598628	7 kDa	15	0.0466	75	0.1508	53	0.1269	40	0.1003	26	0.0579	22	0.0566	29	0.0691
CviNaTBet_HT_01	MT598599	7 kDa	6	2.15%	35	8.12%	19	5.48%	na	na	7	2.70%	na	na	na	na

CviNaTBet_HT_06	MT593315	7 kDa	5	2.07%	11	2.94%	5	1.66%	3	1.05%	16	7.13%	10	3.62%	2	0.79%
CviNaTBet_BCR_10	MT598596	7 kDa	3	1.09%	8	1.88%	5	1.46%	4	1.23%	2	0.78%	11	3.51%	2	0.70%
CviNaTBet_AgSp668	MT598610	7 kDa	3	1.09%	8	1.88%	2	0.58%	4	1.23%	na	na	na	na	1	0.35%
CviNaTBet_HT_06/Na667 like	MT593315	7 kDa	5	2.07%	11	2.94%	5	1.66%	3	1.05%	16	7.13%	10	3.62%	2	0.79%
CviNaTBet_Chin05	MT598606	7 kDa	3	1.09%	8	1.88%	5	1.46%	4	1.23%	2	0.78%	11	3.51%	2	0.70%

Table S5. *C. vittatus* collection sites (USA) and GenBank Accession numbers.

Sequence_ID	Organism	Population variant	Lat_Lon
>CscNaTBet01	*C sculpturatus*	Tempe, AZ	33.36009167, -111.91472222
>CscNaTBet05	*C sculpturatus*	Tempe, AZ	33.36009167, -111.91472222
>CscNaTBet06	*C sculpturatus*	Tempe, AZ	33.36009167, -111.91472222
>CviNaTBet_BCR_01	*C. vittatus*	Big Bend Nat.Pk, TX	29.2000000, -102.9194444
>CviNaTBet_BCR_02	*C. vittatus*	Big Bend Nat.Pk, TX	29.2000000, -102.9194444
>CviNaTBet_BCR_03	*C. vittatus*	Big Bend Nat.Pk, TX	29.2000000, -102.9194444
>CviNaTBet_BCR_04	*C. vittatus*	Big Bend Nat.Pk, TX	29.2000000, -102.9194444
>CviNaTBet_BCR_05	*C. vittatus*	Big Bend Nat.Pk, TX	29.2000000, -102.9194444
>CviNaTBet_BCR_06	*C. vittatus*	Big Bend Nat.Pk, TX	29.2000000, -102.9194444
>CviNaTBet_BCR_07	*C. vittatus*	Big Bend Nat.Pk, TX	29.2000000, -102.9194444
>CviNaTBet_BCR_08	*C. vittatus*	Big Bend Nat.Pk, TX	29.2000000, -102.9194444
>CviNaTBet_BCR_09	*C. vittatus*	Big Bend Nat.Pk, TX	29.2000000, -102.9194444
>CviNaTBet_BCR_10	*C. vittatus*	Big Bend Nat.Pk, TX	29.2000000, -102.9194444
>CviNaTBet_BCR_11	*C. vittatus*	Big Bend Nat.Pk, TX	29.2000000, -102.9194444
>CviNaTBet_BCR_13	*C. vittatus*	Big Bend Nat.Pk, TX	29.2000000, -102.9194444
>CviNaTBet_BCR_14	*C. vittatus*	Big Bend Nat.Pk, TX	29.2000000, -102.9194444
>CviNaTBet_BCR_15	*C. vittatus*	Big Bend Nat.Pk, TX	29.2000000, -102.9194444
>CviNaTBet_HT_01	*C. vittatus*	Hueco Tanks, TX	31.9202778, -106.1216667
>CviNaTBet_HT_02	*C. vittatus*	Hueco Tanks, TX	31.9202778, -106.1216667
>CviNaTBet_HT_03	*C. vittatus*	Hueco Tanks, TX	31.9202778, -106.1216667
>CviNaTBet_HT_04	*C. vittatus*	Hueco Tanks, TX	31.9202778, -106.1216667
>CviNaTBet_HT_05	*C. vittatus*	Hueco Tanks, TX	31.9202778, -106.1216667
>CviNaTBet_HT_06	*C. vittatus*	Hueco Tanks, TX	31.9202778, -106.1216667
>CviNaTBet_HT_07	*C. vittatus*	Hueco Tanks, TX	31.9202778, -106.1216667
>CviNaTBet_HT_08	*C. vittatus*	Hueco Tanks, TX	31.9202778, -106.1216667
>CviNaTBet_HT_09	*C. vittatus*	Hueco Tanks, TX	31.9202778, -106.1216667

>CviNaTBet_Chin_01	*C. vittatus*	Chinati Hot Springs, TX	30.0141667, -104.6880556
>CviNaTBet_Chin_02	*C. vittatus*	Chinati Hot Springs, TX	30.0141667, -104.6880556
>CviNaTBet_Chin_03	*C. vittatus*	Chinati Hot Springs, TX	30.0141667, -104.6880556
>CviNaTBet_Chin_04	*C. vittatus*	Chinati Hot Springs, TX	30.0141667, -104.6880556
>CviNaTBet_Chin_05	*C. vittatus*	Chinati Hot Springs, TX	30.0141667, -104.6880556
>CviNaTBet_Chin654	*C. vittatus*	Chinati Hot Springs, TX	30.0141667, -104.6880556
>CviNaTBet_AgSp_01	*C. vittatus*	Aguirre Springs, NM	32.3872222, -106.5519444
>CviNaTBet_AgSp_02	*C. vittatus*	Aguirre Springs, NM	32.3872222, -106.5519444
>CviNaTBet_AgSp_03	*C. vittatus*	Aguirre Springs, NM	32.3872222, -106.5519444
>CviNaTBet_AgSp_04	*C. vittatus*	Aguirre Springs, NM	32.3872222, -106.5519444
>CviNaTBet_AgSp_05	*C. vittatus*	Aguirre Springs, NM	32.3872222, -106.5519444
>CviNaTBet_AgSp667	*C. vittatus*	Aguirre Springs, NM	32.3872222, -106.5519444
>CviNaTBet_AgSp668	*C. vittatus*	Aguirre Springs, NM	32.3872222, -106.5519444
>CviNaTBet_AgSp_09	*C. vittatus*	Aguirre Springs, NM	32.3872222, -106.5519444
>CviNaTBet_AgSp_11	*C. vittatus*	Aguirre Springs, NM	32.3872222, -106.5519444
>CviNaTBet_AgSp_12	*C. vittatus*	Aguirre Springs, NM	32.3872222, -106.5519444
>CviNaTBet_AgSp_13	*C. vittatus*	Aguirre Springs, NM	32.3872222, -106.5519444
>CviNaTBet_LBR_01	*C. vittatus*	Little Blue River, NE	40.2011111, -097.3883333
>CviNaTBet_LBR_02	*C. vittatus*	Little Blue River, NE	40.2011111, -097.3883333
>CviNaTBet_LBR_03	*C. vittatus*	Little Blue River, NE	40.2011111, -097.3883333
>CviNaTBet_LBR_04	*C. vittatus*	Little Blue River, NE	40.2011111, -097.3883333
>CviNaTBet_LPb_01	*C. vittatus*	Lake Pueblo, CO	38.2683333, -104.7083333
>CviNaTBet_LPb_02	*C. vittatus*	Lake Pueblo, CO	38.2683333, -104.7083333
>CviNaTBet_LPb_03	*C. vittatus*	Lake Pueblo, CO	38.2683333, -104.7083333
>CviNaTBet_LPb_04	*C. vittatus*	Lake Pueblo, CO	38.2683333, -104.7083333
>CviNaTBet_LPb_05	*C. vittatus*	Lake Pueblo, CO	38.2683333, -104.7083333
>CviNaTBet_BMe_01	*C. vittatus*	Black Mesa SP, OK	36.9458333, -102.9430556
>CviNaTBet_PaDu_01	*C. vittatus*	PaloDuro Canyon, TX	34.9966667, -101.9130556
>CviNaTBet_PaDu_02	*C. vittatus*	PaloDuro Canyon, TX	34.9966667, -101.9130556
>CviNaTBet_PaDu_03	*C. vittatus*	PaloDuro Canyon, TX	34.9966667, -101.9130556
>CviNaTBet_PaDu_04	*C. vittatus*	PaloDuro Canyon, TX	34.9966667, -101.9130556
>CviNaTBet_PaDu_05	*C. vittatus*	PaloDuro Canyon, TX	34.9966667, -101.9130556
>CviNaTBet_PaDu_06	*C. vittatus*	PaloDuro Canyon, TX	34.9966667, -101.9130556
>CviNaTBet_Kisa_01	*C. vittatus*	Kisatchie Forest, LA	31.4750000, -092.9988889

>CviNaTBet_Kisa_02	*C. vittatus*	Kisatchie Forest, LA	31.4750000, -092.9988889
>CviNaTBet_Kisa_03	*C. vittatus*	Kisatchie Forest, LA	31.4750000, -092.9988889
>CviNaTBet_Kisa_04	*C. vittatus*	Kisatchie Forest, LA	31.4750000, -092.9988889
>CviNaTBet_Kisa_05	*C. vittatus*	Kisatchie Forest, LA	31.4750000, -092.9988889
>CviNaTBet_KS_01	*C. vittatus*	Lawrence, KS	38.9100000, -095.3286111
>CviNaTBet_KS_02	*C. vittatus*	Lawrence, KS	38.9100000, -095.3286111
>CviNaTBet_KS_03	*C. vittatus*	Lawrence, KS	38.9100000, -095.3286111
>CviNaTBet_KS_04	*C. vittatus*	Lawrence, KS	38.9100000, -095.3286111
>CviNaTBet_KS_05	*C. vittatus*	Lawrence, KS	38.9100000, -095.3286111
>CviNaTBet_Ha1210	*C. vittatus*	Ha Ha Tonka SP	37.9736111, -092.7627778
>CviNaTBet_Scv_01	*C. vittatus*	Scottsville, AR	35.4658333, -093.0400000
>CviNaTBet_Scv_02	*C. vittatus*	Scottsville, AR	35.4658333, -093.0400000

Table S6. NCBI Accession data for rtPCR reference gene and rtPCR primer sequences. Actin rtPCR primers were identified through a *Chilobrachys bowlingi* alpha-tubulin & *C. guangxiensis* cDNA Actin blasted against preliminary genome assembly of *C. vittatus*. .

>gi|28915839|gb|CB334087.1|CB334087 Mg_AFT_28H11_M13F Elongation factor 1-gamma type 1.
>gi|28627174|gb|BU092001.1|BU092001 Mg_AFB_07G10_M13F Elongation factor 2.
>gi|28627232|gb|BU092059.1|BU092059 Mg_AFB_09B10_M13F HISTONE H1.
>gi|28915796|gb|CB334044.1|CB334044 Mg_AFT_28C07_ Ribosomal protein L19
>gi|28915727|gb|CB333975.1|CB333975 Mg_AFT_14G09_M13F Ribosomal protein L19
>gi|28915710|gb|CB333958.1|CB333958 Mg_AFT_14C09_M13F Ribosomal protein L19

Primer Id	Sequence 5′–3′	PCR product size
rtCv_EF2FB	ATGTCCGTTATTGCCCATGT	102 bp
rtCv_EF2RB	TCTCATCTCACCAGCTTTGG	
rtCv_ActinF	TTCCAACCATCCTTCGTGGG	128 bp
rtCv_ActinR	GGTACCACCAGACAAGACGG	
rtCv_ATubulinF	CGCCTTTATGGTCGACAACG	123 bp
rtCv_ATubulinR	GACGCGGTTATGGACGAAAC	
rtCv_EF2F	CTTCACCGTAGACGAGATCC	135 bp
rtCv_EF2R	GCAGCAATGATTCCAGCCTTA G	
rtCv_RPL19F	CTGTGGCTGTTCACTCCCG	102 bp

rtCv_RPL19R	GGGCGTTAGCGGTACCTTTC	
rtCv_Chin654F	CGTGCTGCAAATACACTCGC	125 bp
rtCv_Chin654R	CCATGAATGGGCCAAGTCGG	
rtCv_AgSp_667F	GTTATCTGGCGATCAGGGGC	126 bp
rtCv_ AgSp_667R	GCAGACCTTCGCAATAGCAC	
rtCv_ AgSp_668F	CGGTTGCACGCCCTATTTTG	135 bp
rtCv_ AgSp_668R	GGCCAAGTCGGTGTACTTTC	
Ha1210_F	ATATGGAATGCAAAGCGCCG	122 bp
Ha1210_R	TGCCGCATGATTTACCAGGA	
CsBetaTox_F	AAGGCAAAGAAGGGTTACTGC	89 bp
CsBetaTox_R	TGGTTGCTCTGTCCCAAACTT	
CvIV4_F	TCCAGTTGAAGCAAGTGGTTG	129 bp
CvIV4_R	ACCTGTACACCAACACGACA	
rtCv_HT_09_F	CTGCAAATACGGTTGCACGC	116 bp
rtCv_ HT_09_R	CTGTGCACCAGCATCCGAAA	
rtCv_LPb_03_F	GCTGCAAATACGGTTGCGT	79 bp
rtCv_ LPb_03_R	TTACCACCTTGGTTCGGCG	
